# Evaluation of the INTERGROWTH-21^st^ Neurodevelopment Assessment (INTER-NDA) in 2 year-old children

**DOI:** 10.1371/journal.pone.0193406

**Published:** 2018-02-28

**Authors:** Elizabeth Murray, Michelle Fernandes, Charles R. J. Newton, Amina Abubakar, Stephen H. Kennedy, Jose Villar, Alan Stein

**Affiliations:** 1 Department of Psychiatry, Warneford Hospital, University of Oxford, Oxford, United Kingdom; 2 Nuffield Department of Obstetrics & Gynaecology, John Radcliffe Hospital, University of Oxford, Oxford, United Kingdom; 3 Oxford Maternal & Perinatal Health Institute, Green Templeton College, University of Oxford, Oxford, United Kingdom; 4 Department of Paediatrics, Southampton General Hospital, University of Southampton, Southampton, United Kingdom; 5 Department of Psychology, Lancaster University, Lancaster, United Kingdom; University of Ottawa Faculty of Medicine, CANADA

## Abstract

**Background:**

The INTER-NDA is a novel assessment of early child development measuring cognition, language, motor skills, behaviour, attention, and socio-emotional reactivity in 2 year olds in 15 minutes. Here, we present the results of an evaluation of the INTER-NDA against the Bayley Scales of Infant Development III edition (BSID-III), its sensitivity and specificity and its psychometric properties.

**Methods:**

Eighty-one infants from Oxford, UK, aged 23.1–28.3 months, were evaluated using the INTER-NDA and the BSID-III. The agreement between the INTER-NDA and the BSID-III was assessed using interclass correlations (for absolute agreement), Bland-Altman analyses (for bias and limits of agreement), and sensitivity and specificity analyses (for accuracy). The internal consistency of the INTER-NDA and uni-dimensionality of its subscales were also determined.

**Results:**

The interclass correlation coefficients between the BSID-III and the INTER-NDA cognitive, motor and behaviour scores ranged between 0.745 and 0.883 (p<0.001). The Bland-Altman analysis showed little to no bias in the aforementioned subscales. The sensitivity and specificity of INTER-NDA cognitive scores ≤1 SD below the mean are 66.7% and 98.6% respectively, with moderate agreement between INTER-NDA and BSID-III classifications (κ = 0.72, p<0.001). The sensitivity and specificity of INTER-NDA scores <2 SD below the mean, in predicting low BSID-III scores (<70), are 100% each for cognition, and 25% and 100% respectively for language. More than 97% of children who scored in the normal range of the INTER-NDA (<1SD below mean) also scored in the normal range in the BSID-III (≥85). The INTER-NDA demonstrates satisfactory internal consistency and its subscales demonstrate good unidimensionality.

**Conclusion:**

The INTER-NDA shows good agreement with the BSID-III, and demonstrates satisfactory psychometric properties, for the assessment of ECD at 22–28 months.

## Introduction

The first 1000 days of life, from conception to age 2 years, is the most important period of human neurodevelopment[1–5]. The age of 2 years marks the point at which early child development (ECD) can reliably be assessed because after this period: (i) neurodevelopment is no longer confounded by transient neurological syndromes of prematurity; and (ii) instruments, such as the Bayley Scales of Infant Development (BSID), have good predictive validity in the medium- to long-term[6]. However, ECD assessment at population level depends on: (i) the availability of large numbers of appropriately trained professionals[7, 8]; and (ii) objective assessment tools that can be administered reliably and easily[9]. These often limit steps in population-wide ECD screening and surveillance efforts.

A number of short, resource-light neuropsychological assessments, targeted at 2-year-old children, endeavor to address these requirements and are currently being used in clinical and research settings. These include the Ages and Stages Questionnaire (ASQ)[10], the Brief Infant Toddler-Social and Emotional Assessment (Brief ITSEA)[11], the Child Behavior Checklist (CBCL)[12], the Developmental Milestones Checklist (DMC)[13], the Rapid Neurodevelopment Assessment (RNDA)[14] and the Ten Questions Questionnaire (TQQ)[15]. While some focus on sensitively assessing target areas of neurodevelopment (for example, the CBCL for behavior and the Brief-ITSEA for social and emotional regulation), others (such as the DMC and RNDA) employ a more generalist and culturally appropriate approach. Nevertheless, many evaluations, including ‘gold standard’ assessment tools, are vulnerable to administrative and technical errors [16]. In the BSID-III, for example, it is estimated that these errors occur in approximately 39% of assessments, accounting for inaccuracies in >10% of scores[16].

The challenges for ECD surveillance are: (i) the assessment of multiple dimensions of neurodevelopment, at population-level, with a high degree of reliability, objectivity and precision; and (ii) the suitability of such assessments for international use, without compromising on features essential for scalability (namely short administration time, low costs and relative ease of administration by non-specialists).

To address this issue, The International Fetal and Newborn Growth Consortium for the 21^st^ Century (INTERGROWTH-21^st^) developed, in 2014, an objective, rapid ECD assessment for use by non-specialists, in low-, middle- and high-income countries[17]. Entitled the INTERGROWTH-21^st^ Neurodevelopmental Assessment (INTER-NDA), it assesses cognition, expressive and receptive language, gross and fine motor skills, behaviour, attention and social-emotional reactivity in 2 year olds with an administration time of 15–20 minutes. It consist of 53 directly administered, concurrently observed and caregiver reported items. The INTER-NDA was designed to be free from cultural biases and is based upon objective reporting (rather than subjective judgement) of the child’s performance[17]. Outcomes are reported on a 5-point scale characterising the child’s performance in each domain across a spectrum. The inter-rater and test-retest reliability of the INTER-NDA, determined across 21 assessors in Brazil, India, Italy, Kenya and the UK using Cohen’s kappas, were k = 0.70, 95% CI: 0.47–0.88 and k = 0.79, 95%CI: 0.48–0.96 respectively[17]. The training materials and operation manual for the INTER-NDA are freely available at www.intergrowth21.org.uk/protocol.aspx?lang51).

This report presents the results of a study evaluating the performance of the INTER-NDA against a well-established measure of child development, the BSID-III. The specific aims of the current study were to: (i) evaluate agreement between the INTER-NDA and the BSID-III using interclass correlations and the Bland Altman analyses; (ii) determine the sensitivity and specificity of the INTER-NDA in identifying low scores on the BSID-III; and (iii) determine the internal consistency of the INTER-NDA and the uni-dimensionality of its subscales as an ECD measure.

## Material and methods

### Study population

Eighty-one children (44 boys and 37 girls), with a mean age of 25.4 (*SD* = 1.1) months, were drawn randomly from UK-based families enrolled in the Oxford Postnatal Treatment (OPT) Study, a treatment trial for women with postnatal depression[18], between March 2011 and December 2013. The families resided in Oxfordshire, Buckinghamshire and Berkshire, England. GPs and Health Visitors gave potential participants an overview of the study to and provided a leaflet and contact information. Interested mothers could then telephone, return self-referral forms in the post, or email the study team. Mothers needed to contact the study directly rather than be referred by a healthcare professional. In some instances, following discussion with a mother, a Health Visitor or GP contacted the team on the mother’s behalf. A member of the study team would then contact the mother to assess her eligibility for the study. Women were eligible for inclusion if they met full diagnostic criteria for Major Depressive Disorder (MDD) persistently over the previous 3 months, were ≥18 years old, and their infants were born ≥ 35weeks gestation, had birth weight of ≥ 2000g, were aged 4.5 to 9 months, and had no serious medical conditions. Women were excluded if they were unable to converse in English, suffered from a severe psychiatric diagnosis (other than MDD) or serious physical illness, were not cohabiting with the child, or were currently receiving psychological therapy. The mean maternal age at delivery was 31.80 years (SD = 5.49 years), 91% of mothers (n = 73) had an education level of GSCE (A*-C) and above, and 98% were married or cohabiting (n = 79). The children had a mean birth weight of 3539g (SD = 543g) and a mean gestational age at birth of 39^+6^ weeks (SD = 1^+4^). Further demographic information is presented in S1 Table. The cognitive, expressive communication, receptive communication, overall language and behaviour subscales of the Bayley Scales of Infant Development-Third Edition (BSID-III) were administered at follow-up when the children were approximately two years of age.

### Instruments

#### The BSID-III

The BSID-III is a well-established child development assessment, measuring cognition, language skills, social-emotional skills, motor skills and adaptive behavior from 1 to 42 months. It has a binary (pass/fail) scoring system for each item with assessments continuing until a child fails five consecutive items. This results in a ceiling score, which yields a developmental age for the child. The latter is clinically important in terms of diagnosis, monitoring and evaluating the impact of interventions[19]. The administration time is 60–90 minutes. The cost of the test, at the time of writing this paper, is US$ 1135.00.

The version of the BSID-III used in the OPT study comprised five of the seven subscales (cognitive, expressive communication, receptive communication, overall language and behaviour subscales). Fine and gross motor subscales were not administered as part of the OPT study. In keeping with the BSID-III scoring system, scaled scores (M = 10, SD = 3) were derived for expressive and receptive communication, and composite scores (M = 100, SD = 15) were derived for cognitive and overall language. The total behaviour score was converted to a z-score.

This method of using a subset of subscales for validation has been shown from previous evidence to be an accepted method of validation of a new measure[20, 21]. This is because comparisons are conducted at subscale level, rather than at the level of a singular, global result[20, 21].

#### The INTER-NDA

**Fig 1:**
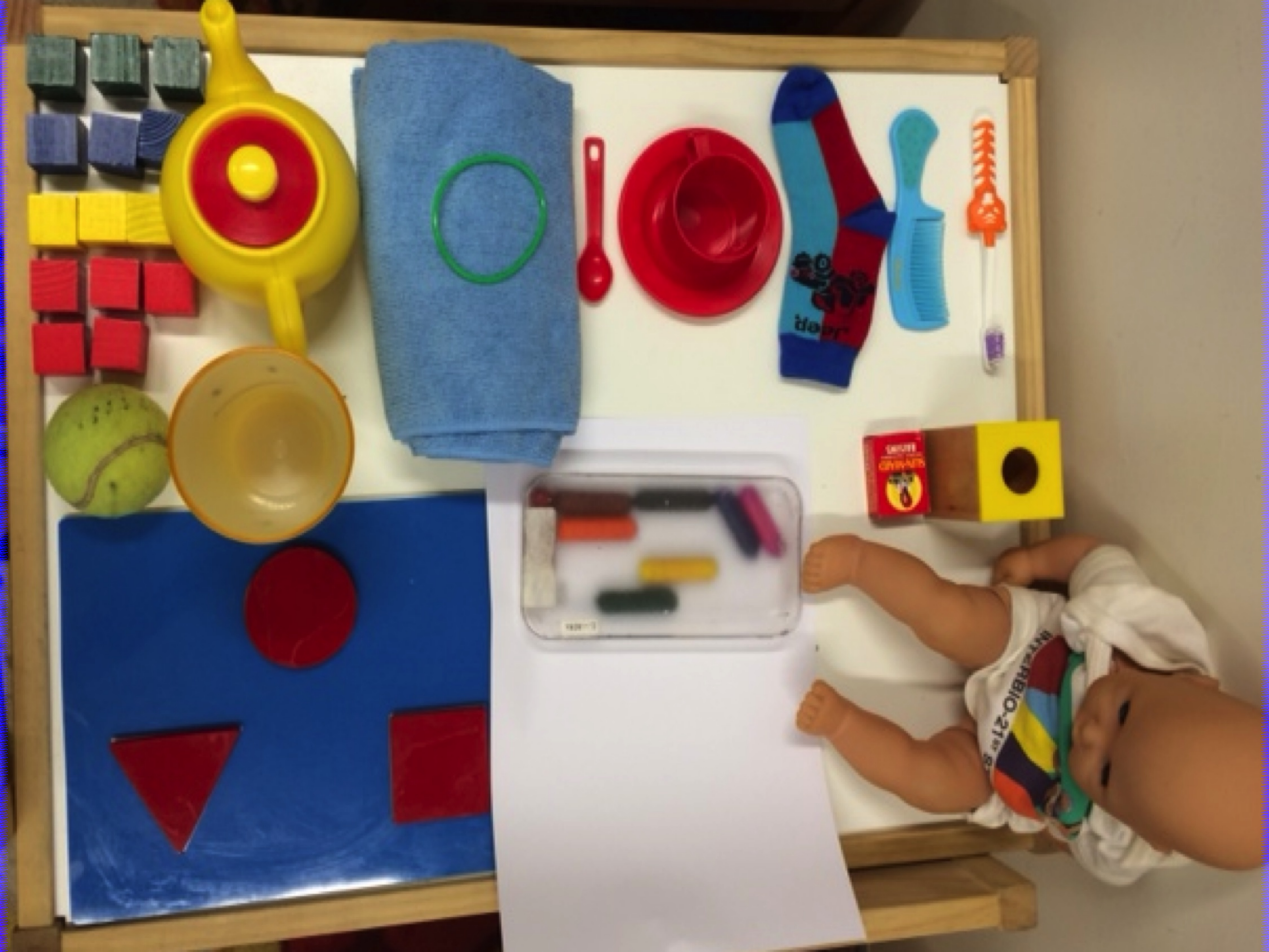
INTER-NDA kit items.

As described above, the INTER-NDA measures cognition, language, motor skills, behavior, attention and social-emotional reactivity in the 22 to 26 month age group[17]. The cost of the INTER-NDA, at the time of writing this paper, is US$ 121.00 (Fig 1).

### Methods

At the time of their second birthday, children enrolled in the OPT study were assessed for cognition, expressive and receptive communication, overall language and behavior on the BSID-III by qualified BSID-III assessors. Assessments were carried out in a clinical research setting with a mean administration time of 60 minutes. All assessments were video-recorded using three cameras at different angles to ensure adequate capture of all aspects of the child’s performance and interaction with the assessor. An independent assessor (EM), trained in the INTER-NDA, reviewed each recording and extracted INTER-NDA cognitive, language and behavior scores (Table 1) for each child from these recordings. The motor, attention and emotional reactivity subscales of the INTER-NDA were not extracted as these subscales were not administered during the BSID-III assessment in the OPT study.

**Table 1:** Scoring system of the INTER-NDA.

Domain	Total Score^	Mean score#	Additional Notes
**Cognition**	Σ (1,2,4,5,6,7,8,11,12,13,14,16,18)	Mean (1,2,4,5,6,7,8,11,12,13,14,16,18)	
**Expressive Language**	Σ (3,7,23,24,25, 26,27,28,29,30)	Mean (3,7,23,24,25, 26,27,28,29,30)	Expressive and receptive language maybe combined to obtain an overall language score
**Receptive Language**	Σ (5,8)	Mean (5,8)	
**Fine Motor**	Σ (9, 10, 15, 20)	Mean (9, 10, 15, 20)	Fine & gross motor maybe combined to obtain an overall motor score
**Gross Motor**	Σ (19, 21, 22)	Mean (19, 21, 22)	
**Positive Behaviour**	Σ (31, 32, 33, 34, 35)	Mean (31, 32, 33, 34, 35)	The difference of mean positive and mean negative behaviour yields a global behaviour score
**Negative Behaviour**	Σ (31, 32, 33, 34, 35)	Mean (31, 32, 33, 34, 35)	
**Attention**	Σ (39, 40, 44, 45, 50)	Mean (39, 40, 44, 45, 50)	Attention
**Social-emotional reactivity**	Σ (38, 41, 42, 43, 46, 47, 48, 49, 51, 52, 53)	Mean (38, 41, 42, 43, 46, 47, 48, 49, 51, 52, 53)	Social Emotional Reactivity

^Total scores are obtained by summing the individual item scores for each domain. The items contributing to each domain are listed in column 1.

#Mean domain scores are the arithmetic mean of all contributing items in a domain scored 1, 2, 3 or 4. Items scored X, i.e. unable to assess, are not included in the calculation of the mean score.

The video-based approach was selected over a conventional 2-session approach (one for the INTER-NDA and one for the BSID-III) to ensure that the child’s scores were not affected by: (i) temporal differences in the child’s mood and rapport with the assessor; or (ii) familiarization with tasks, which may result in higher scores at the second session (given the conceptual overlap between some INTER-NDA and BSID-III items). Furthermore, as the motor subscale of the INTER-NDA overlaps significantly with the motor subscale of the BSID-III, and the attention and social-emotional reactivity subscale of the INTER-NDA overlaps significantly with the Child Behaviour Checklist, it was not considered necessary to perform an evaluation.

### Ethical approval

The OPT study was approved by the Research Ethics Committee (REC ref: 10/H0505/55). The INTERGROWTH-21^st^ Project was approved by the Oxfordshire Research Ethics Committee ‘C’ (reference: 08/H0606/139). In both studies, parents/guardians provided written informed consent on behalf of their children.

### Statistical analysis

#### I. Agreement between the INTER-NDA and the BSID-III

BSID-III and INTER-NDA assessors independently calculated BSID-III and INTER-NDA scores respectively, and were blinded to each other’s calculations. Scaled BSID-III scores (*M* = 10, *SD* = 3) were derived for expressive and receptive communication, and composite scores (*M* = 100, *SD* = 15) were derived for cognitive and overall language. The total behavior score was converted to a z-score. For the INTER-NDA, mean and total scores were calculated for each subscale in accordance with the INTER-NDA scoring system (Table 1), and converted to z-scores.

The total and mean INTER-NDA z-scores for each subscale were assessed against the corresponding BSID-III subscales. Difference scores between BSID-III and INTER-NDA (BSID-III *minus* INTER-NDA) were also calculated.

The agreement between the INTER-NDA and the BSID-III was evaluated using four statistical methods, as recommended by Lee [22] and Bland and Altman[23]: (i) repeated measures *t*-tests to assess whether there was a difference between INTER-NDA and BSID-III scores within subjects; (ii) single measure interclass correlation coefficients (ICCs) for absolute agreement for each subscale, using a two-way mixed effects model (to quantify the strength of the association between INTER-NDA and BSID-III scores); (iii) bias and limits of agreement statistics; and (iv) Bland-Altman plots to identify whether the INTER-NDA scores differed systematically across different levels of the BSID-III, and linear regression analyses of the relationship between difference score and BSID-III score[24].

#### II. Sensitivity and specificity analysis

To assess the accuracy of the INTER-NDA in determining low scores on the BSID-III, INTER-NDA cores were dichotomized into low (≤1SD) and normal (>1SD) and BSID-III scores were dichotomized as borderline (≤85) and normal (>85). Accuracy was assessed using sensitivity and specificity analyses to determine the ability of low INTER-NDA scores to predict borderline composite BSID-III scores. Cohen’s kappa was used to determine the level of agreement between INTER-NDA and BSID-III classifications.

#### III. Internal consistency and uni-dimensionality of the INTER-NDA

Cronbach’s alphas[25] were calculated for each INTER-NDA subscale. Cronbach’s alpha values are considered “good” if they were above a threshold of 0.7[26]. In conceptualizing the INTER-NDA a key consideration was that all the items measure an underlying construct i.e. ‘neurodevelopment at 2 years of age’. To evaluate the extent to which this is true the unidimensionality of scales was evaluated using a confirmatory factor analysis (CFA) in STATA 15. Comparative Fit, chi square, Tuckler Lewis and Root Mean Sqaure Error of Approximation indices were selected to test the CFA model that best represented the data.

All INTER-NDA subscales are presented for mean (and not total) INTER-NDA scores, unless expressly stated otherwise. Mean INTER-NDA subscale scores were selected over total INTER-NDA subscale scores because the former are not affected by external factors (such as the caregiver interfering in the assessment, a sudden distracting influence in the assessment room, or the assessor being aware that he/she has made an error in the task administration). In such situations, the assessor would score the child as ‘X’ (i.e., unable to assess) for that item. This item would be reflected in the total score but not the mean score.

## Results

### I. Agreement between the INTER-NDA and BSID-III

The mean INTER-NDA and BSID scores for the domains of cognition, receptive language/communication, expressive language/communication, overall language, behavior, positive behavior and negative behavior are presented in Table 2. The results of the four statistical approaches are:

**Table 2:** Mean subscale scores, and subscale score comparisons between the INTER-NDA and BSID-III.

Subscale	INTER-NDA Mean (SD)	BSID-III Mean (SD)	Within-subjects t-tests
Cognitive	36.8 (6.5)	98.8 (13.1)	*t*(80) = 0.88, *p* = 0.38
Receptive language/communication	6.2 (1.2)	10.0 (3.0)	*t*(80) = 0.00, *p* = 1.00
Expressive language/communication	31.0 (6.6)	10.2 (3.3)	*t*(80) = 0.00, *p* = 1.00
Overall language	37.2 (7.3)	100.9 (17.3)	*t*(80) = 1.08, *p* = 0.28
Global Behaviour	17.5 (3.4)	33.5 (4.6)	*t*(80) = 0.96, *p* = 0.34
Positive behaviour	12.5 (2.5)	23.0 (3.4)	*t*(80) = -0.13, *p* = 0.99
Negative behaviour	4.9 (1.1)	10.5 (1.4)	*t*(80) = -0.01, *p* = 0.99

**Fig 2:**
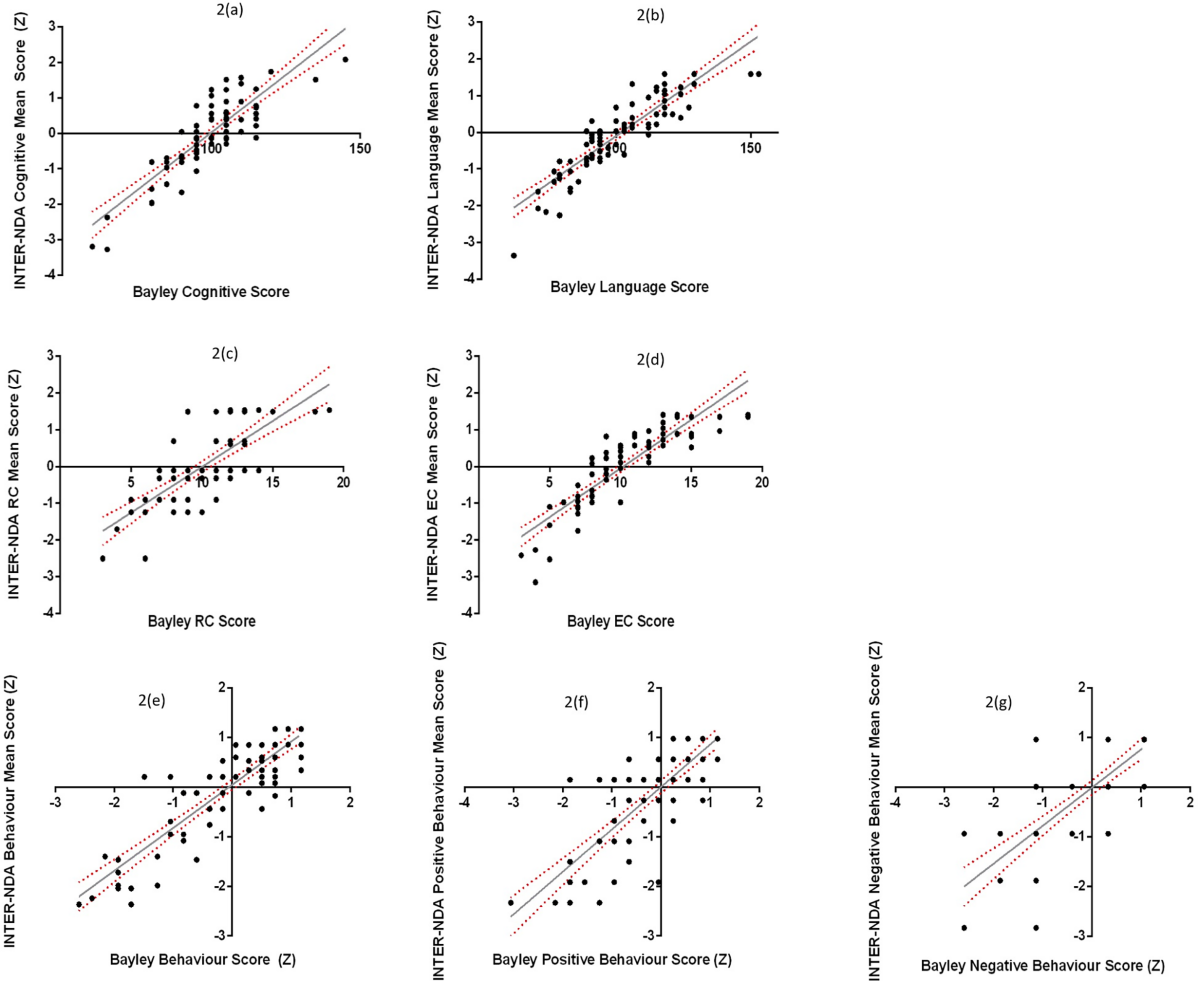
Association between INTER-NDA and BSID-III scores.

**Fig 3:**
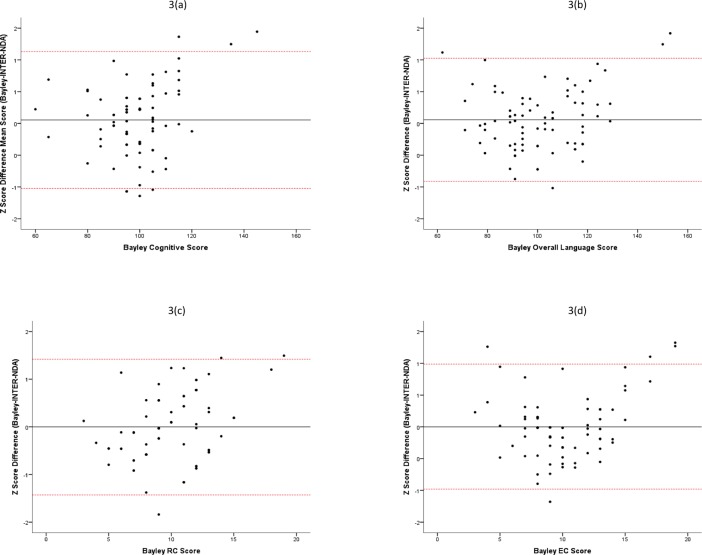
Bland Altman Plots.

1. Repeated measures *t*-tests showed no significant differences in BSID-III and INTER-NDA scores across all subscales (Table 2).
2. The ICCs for the BSID-III and INTER-NDA subscales (Table 3) indicate a strong association between all INTER-NDA subscales and BSID-III equivalents. All INTER-NDA subscales are within the limit of acceptability for the lower limit of the ICC confidence interval proposed by Bland & Altman (not <0.75)[23, 24]. Fig 2 depicts the association between BSID-III scores and INTER-NDA z-scores across the subscales.
3. The Bland-Altman analysis indicated no, or very low, bias in the subscales (Table 4), suggesting very little difference between INTER-NDA and BSID-III scores[23].
4. The Bland-Altman plots and linear regression analyses of the difference scores (BSID-III *minus* INTER-NDA) revealed positive associations between the subscales (Table 5; Fig 3) such that variation in the BSID-III score accounted for 7.2%, 13.4%, 6.7%, 5.1%, 7.6%, 6.6% and 12.2% of the difference between the BSID-III and INTER-NDA score in the cognitive, receptive language, expressive language, overall language, total behavior, positive behavior and negative behavior subscales, respectively.

**Table 3:** Interclass correlation coefficients between the INTER-NDA and BSID-III subscales.

Subscale	Interclass correlation coefficient (95% CI)
Cognitive	0.85 (0.77–0.90)**
Receptive language/communication	0.74 (0.63–0.83)**
Expressive language/communication	0.88 (0.82–0.92)**
Overall language	0.88 (0.83–0.92)**
Global Behaviour	0.88 (0.82–0.92)**
Positive behaviour	0.84 (0.77–0.90)**
Negative behaviour	0.78 (0.67–0.85)**

**p < .001

**Table 4:** Bland-Altman analysis showing agreement between the BSID-III and the INTER-NDA.

Subscale	Bland-Altman analysis		
	Bias	Lower limit of agreement	Upper limit of agreement
Cognitive	0.05	-1.02	1.13
Receptive language/communication	0.00	-1.40	1.40
Expressive language/communication	0.00	-0.96	0.96
Overall Language	0.06	-0.89	1.01
Global Behaviour	-0.05	-1.03	0.92
Positive behaviour	-0.01	-1.13	1.11
Negative behaviour	0.00	-1.34	1.34

**Table 5:** Difference scores between the BSID-III and the INTER-NDA.

Subscale	Linear regression (difference scores vs BSID-III scores)	
	*r*	*p*
Cognitive	0.27	0.02
Receptive language/communication	0.37	0.001
Expressive language/communication	0.26	0.02
Overall language	0.23	0.04
Global behaviour	0.28	0.01
Positive behaviour	0.26	0.02
Negative behaviour	0.35	0.001

### II. Sensitivity and specificity analysis

The results of this analysis are presented in Table 6. The sensitivity and specificity of INTER-NDA cognitive scores <2 SD below the mean, for determining low BSID-III scores (<70), are 100% respectively, with strong agreement between INTER-NDA and BSID-III classifications (κ = 1.00, p<0.001). The sensitivity and specificity of INTER-NDA language scores <2 SD below the mean, for determining low BSID-III scores (<70), are 25.0% and 100% respectively, with fair agreement between INTER-NDA and BSID-III classifications (κ = 0.39, p<0.05).

**Table 6:** Prevalence of low cognitive and language scores on the INTER-NDA (<2SD) and BSID-III (<70): Sensitivity, specificity and agreement between scales (Cohen’s kappa).

INTER-NDA	BSID-III <70	BSID-III≥70	Sensitivity	Specificity	Κ (p)
**Cognitive**					
<2SD	3	0	100.0%	100.0%	1.00 (<0.05)
≥2SD	0	78			
**Overall language**					
<2SD	1	3	25.0%	100.0%	0.39 (<0.001)
≥2SD	0	77			

### III. Internal consistency and uni-dimensionality of the INTER-NDA

The Cronbach’s alpha scores are presented in Table 7. These were good for the cognitive, receptive language, expressive language, and positive behaviour subscales of the INTER-NDA and was acceptable for the negative behaviour subscale[26]. The corresponding internal consistencies of the BSID-III, calculated from the OPT data, are also presented in the table for comparison.

**Table 7:** Internal consistencies of the INTER-NDA and the BSID-III.

Subscale	Cronbach’s alphas	
	INTER-NDA	Bayley-III
Cognitive	0.81	0.88
Receptive language/communication	0.83	0.89
Expressive language/communication	0.90	0.93
Positive Behaviour	0.85	0.82
Negative Behaviour	0.56	0.62

**Fig 4:**
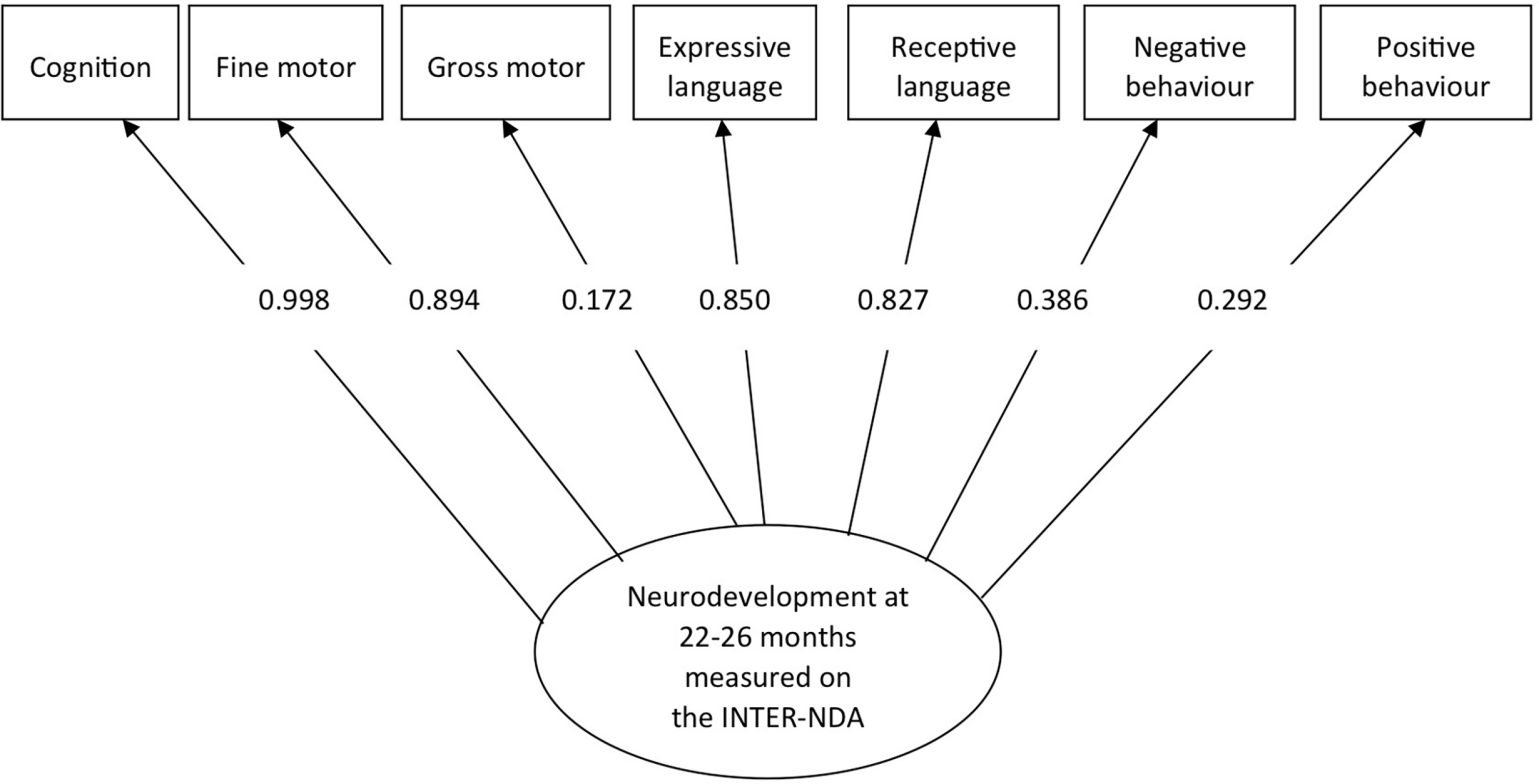
Confirmatory factor analysis of INTER-NDA subscales.

The seven subscales of the INTER-NDA (cognition, receptive language, expressive language, fine motor, gross motor, positive behaviour and negative behaviour) showed a good fit to the model since all the fit indices were above the recommended values with the exception of Root Mean Square Error of Approximation: The fit indices were: Comparative Fit Index = 0.90 (recommended: >0.90); χ^2^ = 66.52, *p* < .001; Tuckler Lewis Index = 0 .94 (recommended: >0 .90, and Root Mean Square Error of Approximation = 0.16 (recommended: <0.80). Fig 4 presents the standardized coefficients from the loadings.

## Discussion

The INTER-NDA is a multi-dimensional ECD instrument measuring cognition, language, motor skills, behavior, attention and social-emotional reactivity in the 22–26 month age group. It has been used to assess neurodevelopment in children from the INTERGROWTH-21^st^ Project sites in Brazil, India, Italy, Kenya and the UK. In this paper, we demonstrate that: (i) it has substantial agreement with the BSID-III for children in the 22–26 month age group; (ii) its sensitivity and specificity for determining low BSID-III scores for the cognitive and language domains are 100%; and 25% and 100% respectively; and (iii) it demonstrates satisfactory internal consistency and its subscales demonstrate good uni-dimensionality in contributing to neurodevelopment at 22–28 months.

The results of our evaluation of the agreement between the INTER-NDA and BSID-III show no significant differences between scores for the same child, and substantial associations between the subscales of the two measures. Importantly, the Bland-Altman plots and linear regression analyses (Fig 3; Tables 5 and 6) reveal that, for the extremely low scores of the BSID-III, children scored relatively higher on the INTER-NDA (floor effect) and, where children scored extremely highly on the BSID-III, they scored relatively lower on the INTER-NDA (ceiling effect). This finding reflects the differences in the age range that the tests were designed to assess, i.e. the INTER-NDA for 22–26 months and BSID-III for 1–42 months. It confirms that the INTER-NDA functions well in agreement with the BSID-III within the age group for which it was designed. Moreover, the results of the Bland-Altman analysis confirm the agreement between the INTER-NDA and BSID-III by showing that a child with a mean cognitive score on the INTER-NDA is likely to score 101 on the cognitive composite score of the BSID-III, with a 95% probability that the true value of their score will fall in the range of 85–118. Given that the SD of the BSID-III composite score is 15, the results indicate that 95% of the children scoring at the mean of the INTER-NDA will fall within 1SD below and just over 1SD above the BSID-III mean, evidencing that the two scales are functioning similarly from a statistical and distributional point of view. These results indicate that the likelihood of a child scoring >±1SD of the BSID-III mean, when he/she achieves a mean score on the INTER-NDA, is statistically negligible. We have also shown that the internal consistency of the INTER-NDA subscales is good to acceptable, and comparable with the BSID-III, even though the INTER-NDA has five times fewer items than the BSID-III, and a fifth of the BSID-III’s administration time.

Our study was limited in that all children were UK-based, despite the INTER-NDA being designed for international use. It was also limited in that the agreement between the BSID-III and the INTER-NDA for the gross and fine motor domains could not be ascertained as these subscales were not administered in the OPT Study. Furthermore, the INTER-NDA was scored using video-recordings of the BSID-III and not in real-time. Nevertheless, there are some strengths to using the video-recording evaluation design over the conventional 2-session evaluation approach: first, as the child did not have two separate assessment sessions, changes in the child’s mood, and his/her familiarization with items, were less likely to confound scores. Second, the design permitted BSID-III and INTER-NDA assessors to score the child’s performance on the respective scales independently, without the presence of multiple examiners in the assessment room, but effectively during the same assessment. Third, in essence, each child completed the INTER-NDA and BSID-III assessments at exactly the same age and time, with the same level of rapport with their mother and assessor. Fourth is that the children were drawn from the OPT trial and therefore were exposed to maternal postnatal depression at some stage during their infancy (assessment was conducted at the end of the treatment trial), which in itself might bias the sample. However, it was unlikely that this would affect the validation results, as low-scorers on the BSID-III might be expected to score low on the INTER-NDA as well. Finally, estimates of accuracy (sensitivity and specificity) are based on the underlying assumption that the reference standard is 100% sensitive[27]. Although the Mental Development Index of the BSID has demonstrated moderate sensitivity (57%) and high specificity (90–100%), and is considered the most comprehensive assessment available for infant neurodevelopment[28]; it does have a degree of imperfection, which must be considered when interpreting results[16].

Despite these limitations, this study provides evidence of the agreement between the INTER-NDA and BSID-III in assessing cognitive, language and behavioural components of early childhood development in the 22–28 month age group. This is important because the motivation behind the development of the INTER-NDA was to overcome the dependence on time and infrastructure in the context of ECD measurement, by providing a validated, objective assessment that is rapid, reliable and easy to administer in high-, middle- and low-income settings. The use of such a measure might provide a useful, scalable solution for population-based ECD assessments by shifting the emphasis of delivery channels from time-intensive in-depth assessments, to rapid, easy to administer assessment tools. The INTER-NDA is currently in use in the INTERGROWTH-21^st^ Project study sites in Brazil, India, Italy, Kenya and the UK where more than 1000 children have been assessed to date[8]. In this study, the INTER-NDA has therefore proved to be a satisfactorily valid measure of ECD. Nevertheless, its discriminant and predictive validity, and its potential to be used as a diagnostic ECD measure, remain to be explored. Confirmatory validation across larger sample sizes, and in different socio-geographical contexts, is also needed.

### Conclusion

The INTER-NDA shows good agreement with the BSID-III. It is in use in the INTERGROWTH-21^st^ Project sites in Brazil, India, Italy, Kenya and the UK and may provide a relatively quick, cost-effective solution to holistic population-based ECD measurement.

## Supporting information

S1 Table OPT study sample characteristics. (DOCX)
