# Supplementary material for: Evaluation of the INTERGROWTH-21st Neurodevelopment Assessment (INTER-NDA) in 2 year-old children
Source: PLoS One. 2018 Feb 28;13(2):e0193406. doi: 10.1371/journal.pone.0193406 (PMC5831101; doi:10.1371/journal.pone.0193406)
Supplement: S1 Table — (DOCX) [file pone.0193406.s001.docx]

Table S1 OPT Sample Characteristics

| **Variable (N)** | **n** | **%** | **Variable (N)** | **n** | | **%** |
| --- | --- | --- | --- | --- | --- | --- |
| **Maternal education (80)** | | | **Parents co-reside (80)** | | | |
| <10 years | 2 | 2.5 | Yes | 69 | | 86.3 |
| 10 years (Grade D-G) | 4 | 5.0 | No | 11 | | 13.8 |
| 10 years (Grade A*-C) | 18 | 22.5 | **Partner education (75)** | | | |
| <12 years | 8 | 10.0 | <10 years | 2 | | 2.7 |
| Professional diploma | 4 | 5.0 | 10 years (Grade D-G) | | 4 | 5.3 |
| Foundation degree | 6 | 7.5 | 10 years (Grade A*-C) | 12 | | 16.0 |
| Undergraduate degree | 23 | 28.7 | <12 years | 19 | | 25.3 |
| Masters degree | 10 | 12.5 | Professional diploma | 0 | | 0.0 |
| Doctoral degree | 5 | 6.3 | Foundation degree | 5 | | 6.7 |
| **Marital status (80)** | | | Undergraduate degree | 18 | | 24.0 |
| Single | 6 | 7.5 | Masters degree | 7 | | 9.3 |
| Relationship –non-cohabiting | 1 | 1.3 | Doctoral degree | 8 | | 10.7 |
| Relationship –cohabiting | 73 | 91.3 | **Parity (79)** | | | |
| **Maternal employment (80)** | | | 1 | 40 | | 50.6 |
| Unemployed | 26 | 32.5 | >1 | | 39 | 49.4 |
| Student | 1 | 1.3 | **Child Sex (81)** | | | |
| Maternity leave | 47 | 58.8 | Male | 44 | | 54.3 |
| Part time | 4 | 5.0 | Female | | 37 | 45.7 |
| Full time | 2 | 2.5 | **Admitted to Neonatal Unit (80)** | | | |
| **Parents co-reside (80)** | | | Yes | 4 | | 5.0 |
| Yes | 69 | 86.3 | No | 76 | | 95.0 |
| No | 11 | 13.8 |  |  | |  |
